# Differences in Covid-19 deaths amongst cancer patients and possible mediators for this relationship

**DOI:** 10.1038/s41598-025-95037-3

**Published:** 2025-03-26

**Authors:** Leah Vaidya, Nubaira Rizvi, Xiao-Cheng Wu, Lauren S. Maniscalco, Yong Yi, Augusto Ochoa, Qingzhao Yu

**Affiliations:** 1 Biostatistics and Data Science, School of Public Health, Louisiana State University Health Sciences Center, New Orleans, LA USA; 2 Louisiana Tumor Registry, School of Public Health, Louisiana State University Health Sciences Center, New Orleans, LA USA; 3 Stanley S. Scott Cancer Center, School of Medicine, Louisiana State University Health Sciences Center, New Orleans, LA USA

**Keywords:** Mediation analysis, Covid-19, Cancer, Charlson comorbidity index, Racial difference, Renal disease, Biomarkers, Oncology, Risk factors

## Abstract

Previous research demonstrated Non-Hispanic Black populations experience higher COVID-19 mortality rates than Non-Hispanic White individuals. Additionally, cancer status is a known risk factor for COVID-19 death. While prior studies investigated comorbidities as exploratory variables in differences in COVID-19 hospitalization, none have explored their role in COVID-19-related deaths. This study aimed to evaluate whether Charlson Comorbidity Index (CCI) and subsequently, individual diseases are potential explanatory variables for this relationship. The analysis focused on Non-Hispanic Black and Non-Hispanic White cancer patients aged 20 or older, diagnosed between 2011 and 2019, who tested positive for COVID-19 from the start of pandemic through June 30, 2021 from Louisiana Tumor Registry. Two separate mediation analyses were conducted. First checked whether overall comorbidity, measured by CCI, could explain the difference in COVID-19 mortality. If so, further checked which individual comorbidities contributed to this difference. The hazard rate for Non-Hispanic Black cancer patients dying from COVID-19 was 6.46 times than that of Non-Hispanic White patients. The CCI accounted for 12.7% of the differences observed in COVID-19 mortality, with renal disease as the top contributor, explaining 4.9%. These findings could help develop interventions to reduce COVID-19 mortality and address the disproportionate impact, especially by managing chronic conditions like renal disease.

**Supplementary Information:**

The online version contains supplementary material available at 10.1038/s41598-025-95037-3.

## Introduction

From the onset of the initial outbreaks of the SARS-CoV-2 coronavirus disease 2019 (COVID-19) in late 2019, extending into early 2020, considerable research efforts have focused on investigating potential risk factors associated with the disease. Studies have shown that certain underrepresented groups have an increased risk of COVID-19 infection, hospitalization, and death. In particular, studies examining race as a potential predictor for COVID-19 outcomes have revealed significantly higher rates of COVID-19 infection^1–5^, hospitalization^1–3,6−8^, and death^9−14^ among Non-Hispanic Black patients compared to Non-Black patients.

Beyond only assessing the existence of differences in health outcomes, we aim to explore potential explanatory variables for this relationship. Previous studies have shown a relationship between age^1,4,6–8,17–19^, sex^1,4,6,7,8,17–18,20^ and COVID-19 outcomes. Furthermore, there is evidence linking race and COVID-19 outcomes to the Charlson Comorbidity Index. This Index predicts 10-year survival in patients based on multiple comorbidities, with studies indicating that Non-Hispanic Black patients tend to have higher Charlson scores. Moreover, individuals with more comorbidities tend to experience worse COVID-19 outcomes^21–23^. In addition, specific diseases exhibit associations with both race and COVID-19-related deaths, with Non-Hispanic Black patients demonstrating higher rates of the disease and individuals with these conditions experiencing elevated COVID-19-related deaths. Conditions such as Myocardial Infarction^24,25^, Chronic Heart Failure^26,27^, Peripheral Vascular Disease^28,29^, Diabetes^30,31^, Renal Disease^32,33^, and Liver Disease^34,35^ are among those linked to increased COVID-19-related deaths. Interestingly, although Chronic Pulmonary Disease patients have higher COVID-19-related deaths, this condition is actually more prevalent in Non-Hispanic White patients^36,37^. Additional demographic variables such as poverty, cancer type, obesity, and insurance were also investigated. Obesity is associated with increased COVID-19 mortality rates^39,40^. Patients with Medicare displayed higher COVID-19 death rates than those with commercial or self-pay insurance^41,42^.

Moreover, poverty was found to be related to COVID-19 death rates, with individuals further below the poverty line exhibiting worse COVID outcomes^38^. Research has shown that individuals living in poverty-stricken neighborhoods face higher risks for chronic diseases, reduced access to healthcare, and worse health outcomes overall^56,62^. Poverty rates have especially exacerbated during the COVID-19 pandemic, with health differences increasing among underrepresented groups^57^. Lower accessibility to health care, exposure to high-risk occupations, and poor neighborhood and housing conditions contributed significantly towards the disproportionate impact of disease^57–59^. During the pandemic, census-tract with higher proportion of poverty experienced significantly higher hospitalization rates after adjusting for other demographic and socio-economic factors^63^.

Underlying these factors are deeply rooted in systemic and institutionalized policies, practices, and societal structures that are detrimental for marginalized communities^58,60^. Especially, historical policies, including segregation and redlining, have systematically excluded Black communities, contributing to the deepening of health differences and worsening overall health outcomes^58^. They were systematically denied opportunities for homeownership and wealth accumulation, which resulted in enduring poverty and poor living conditions that adversely affect health outcomes. These communities also often live in environments with higher rates of comorbidities, such as hypertension, diabetes, and obesity, which are risk factors for severe COVID-19 outcomes^61^.

Additionally, cancer patients, who make up a particularly immunocompromised population, have been identified to have an increased risk of severe COVID-19, ICU admission, and death^15,16^. Research has shown that Non-Hispanic Black cancer patients have higher risk and more severe COVID-19 outcomes than Non-Hispanic White cancer patients^48,49^. While some studies have explored this relationship, further research is necessary to understand the factors that may potentially explain the differences observed in COVID-19 related outcomes for cancer patients.

A previous study investigated the relationship between COVID-19 hospitalization and race among cancer patients, examining various chronic diseases and demographic variables as potential mediators for this relationship^43^. However, this effect has not been examined for COVID-19-related deaths. The aim of this study is to investigate the relationship between COVID-19-related deaths and race amongst cancer patients and examine whether the Charlson Comorbidity Index explains the differences in COVID-19-related death using novel mediation analysis method. We are also interested in exploring which individual disease, in particular, serves as a potential explanatory variable for this relationship. This research is important because it helps us understand further the factors underlying racial differences in COVID-19-related deaths. By identifying these factors, future research and intervention can be tailored to address various determinants, ultimately working toward reducing COVID-19-related deaths and the racial gap among cancer patients.

## Methods

### Data sources

The Louisiana Tumor Registry (LTR) collected cancer and COVID information. LTR is a population-based state cancer registry supported by the National Cancer Institute’s Surveillance, Epidemiology, and End Results (SEER) Program and the National Program of Cancer Registries (NPCR) of the Centers for Disease Control and Prevention. All methods were carried out in accordance with relevant guidelines and regulations. The protocols for this study were reviewed and approved by the LSU Health-New Orleans Institutional Review Board (IRB), ensuring compliance with all ethical standards. Informed consent was obtained from all subjects involved in the study, or their legal guardians where applicable, prior to participation. The variables collected from the LTR database include demographic and clinical variables such as sex, poverty, BMI, insurance, cancer type, summary stage, and the Charlson Comorbidity index. LTR also collects information on patients’ vital status and the cause of death, including COVID-19 as a cause of death. The researchers for this study obtained de-identified dataset only.

The Louisiana statewide COVID-19 data was collected from the Louisiana Department of Health’s (LDH) COVID-19 database. It identified patients who tested positive for COVID-19 by RT-PCR or antigen test. LTR then linked the data from these two databases.

### Inclusion and exclusion criteria

This study focused exclusively on comparing only Non-Hispanic Black and Non-Hispanic White cancer patients. Patients of other racial groups were not included in the analysis due to the limited number of individuals with both cancer and COVID-19 in Louisiana. Race data were predominantly collected from medical records by the Louisiana Tumor Registry, which relies on self-reported measures. This study included cancer patients who were diagnosed between 2011 and 2019 and were 20 years or older. In addition, only patients who tested positive for COVID-19 from the beginning of the pandemic in 2020 through June 30, 2021, were included in the study. This timeframe was chosen because the available cause of death variable was extended until December 31, 2021. Therefore, only cancer patients diagnosed with COVID six months prior to the final recorded death date were included in the study. The cutoff of six months before the final recorded death date was to account for delayed cause of death due to COVID-19.

### Outcome variable

The outcome variable in the analysis was time-to-event, where the event is the death of COVID-19, and the length of time is the time of diagnosis with COVID-19 until the death or last date of contact. COVID-19 cause of death was identified using ICD-10-CM code U071 (https://www.icd10data.com/ICD10CM/Codes/U00-U85/U00-U49/U07-/U07.1). Only cases where the primary cause of death was COVID-19 were counted as events. The death data was distracted from the state death certificate database and the National Death Index. The Social Security Administration database was used to supplement the date of last contact.

### Predictor variable

The predictor variable used in this study was race, classified as Non-Hispanic White patients or Non-Hispanic Black patients.

### Mediators and covariates

**Fig. 1:**
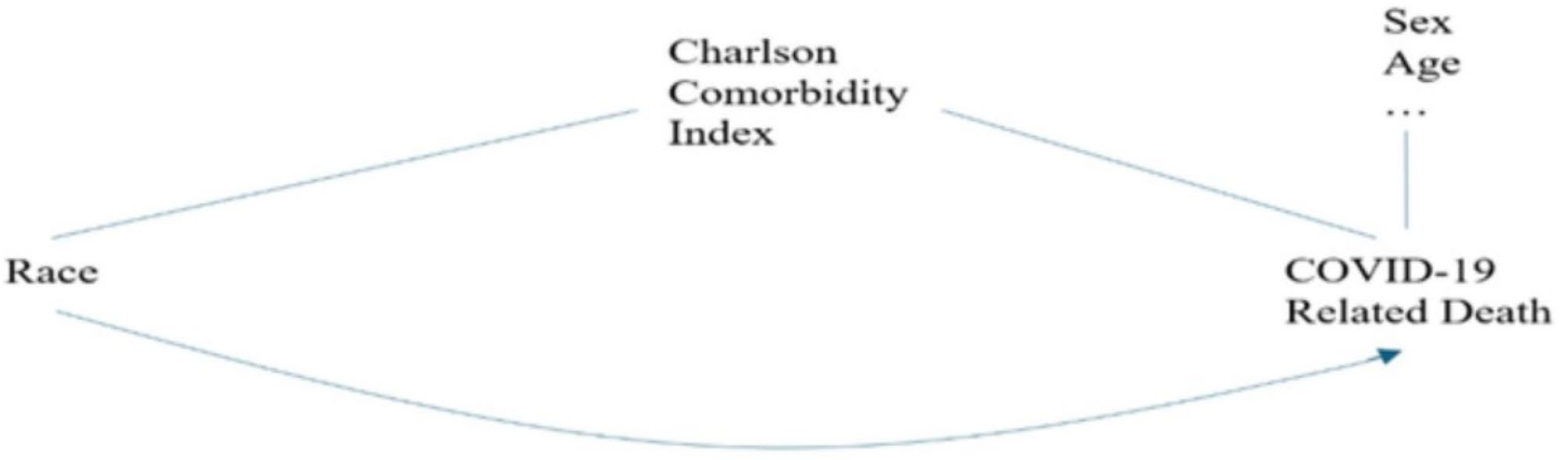
Conceptual model to explore racial difference in COVID-19 mortality for Analysis 1.

Mediators were variables that could explain the relationship between race and the risk of COVID-19 death. Based on previous research, potential mediators were identified by exploring factors that had a relationship associated with both the predictor (race) and the outcome (COVID-19 death) variables. The primary mediator in this study was the Charlson Comorbidity Index, which was coded as 0, 1–2, or 3 + depending on the number of comorbidities. It is a validated scoring system used to predict mortality risk and disease burden by accounting for comorbid conditions in patients. The index includes a total of 19 different comorbidities. Figure 1 shows the conceptual model for this.

**Fig. 2:**
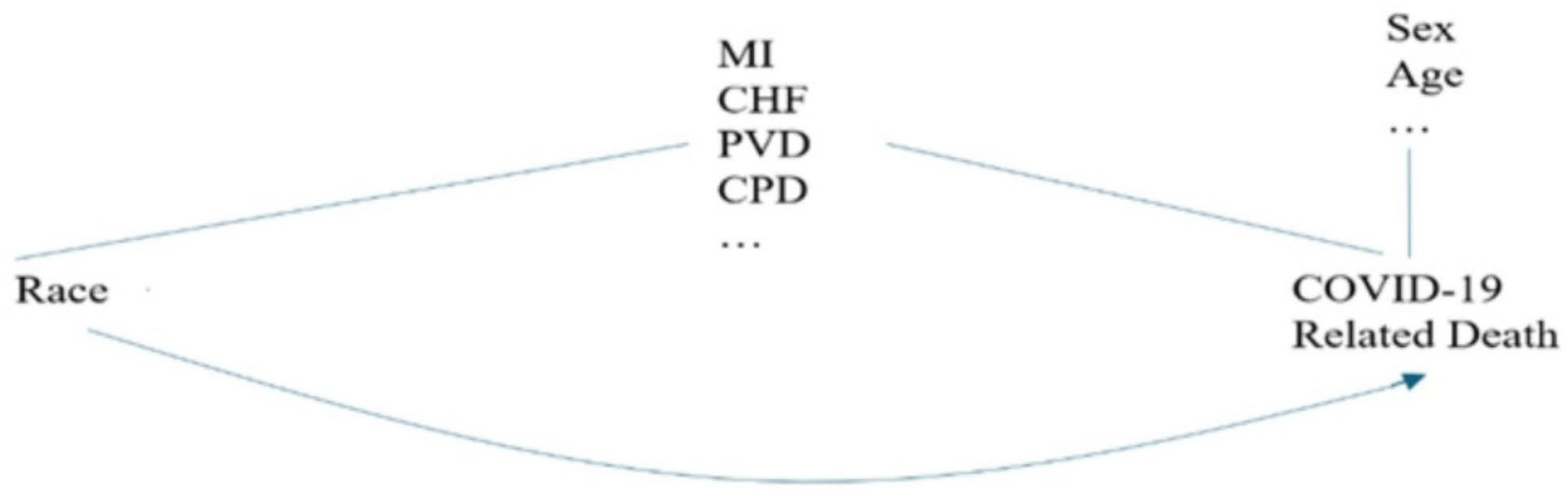
Conceptual model to explore racial difference in COVID-19 mortality for Analysis 2.

In a second analysis, we included individual comorbidity to identify specific diseases that could explain the relationship between race and COVID-19 death. The conceptual model for this is shown in Fig. 2.

The diseases included are listed below in Table 1. All diseases were categorized as Yes (1), having the disease, or No (0), not having the disease. We combined the comorbidities included in the Charlson Index but had a small number of cases into one group of other diseases (dementia, paralysis, ulcers, rheumatic disease, AIDs, and cerebrovascular disease). For other diseases, a patient was categorized as Yes (1) if they had any of the diseases or No (0) if they had none of the diseases. In addition, we also assessed demographic factors (listed in Table 1) that potentially explain the observed differences.

**Table 1:** List of all mediators/covariates included within the two analyses.

Group	Mediator or covariate?	Values
Comorbidity index	Mediator	Charlson Comorbidity Index (0, 1–2, 3 + comorbidities)
Diseases	Mediators	Myocardial Infarction (yes, no)
		Congestive Heart Failure (yes, no)
		Peripheral Vascular Disease (yes, no)
		Chronic Pulmonary Disease (yes, no)
		Diabetes (yes, no)
		Chronic Renal Disease (yes, no)
		Chronic Liver Disease (yes, no)
		Other diseases (yes, no)
Demographic	Covariates	Insurance (private, Medicare/other public, Medicaid, none/unknown)
		Poverty (0%–<10%, 10%–<20%, ≥ 20%, unknown)
		Cancer Type (breast, prostate, colon and rectum, hematopoietic, lung/bronchus, other)
		SEER Summary Stage (in situ, localized, regional, distant, unknown)
		Obesity (yes, no)
		Age at COVID Diagnosis (20–49, 50–64, 65–74, 75+)
		Sex (male, female)

To account for their confounding effects, the variables insurance, poverty, cancer type, SEER summary stage, obesity, age, and sex were all included in the analysis as covariates. All these variables were associated with COVID-19-related death. The insurance variable included in the model pertains to the insurance coverage at the time of cancer diagnosis and the first course of treatment. In the case where a patient has both private and Medicare insurance, they were categorized as having private insurance. The poverty variable categories were determined based on the percentage of individuals with an income below the federal poverty level at the census tract where the patients resided at COVID-19 diagnosis. Cancer type was categorized based on the type of cancer the patient was initially diagnosed with. SEER summary stage is a way of categorizing how far a cancer has spread from its origin. The cancer types were selected based on their prevalence in the study population and their potential impact on survivorship outcomes. Lung cancer was specifically included because COVID-19 primarily affects the respiratory system, and individuals with lung cancer may have heightened vulnerability to severe outcomes.

Additionally, combining the rest of the cancer types in the analysis was done because the direction of the associations for these cancers was similar. In situ is defined as the presence of malignant cells within the cell group from which they arose. Localized cancer refers to cancer that has not spread beyond the boundary or site of origin. Regional indicates tumor extension beyond the site of origin and distant are tumor cells that have grown at a new location. Obesity was classified based on patients’ BMI and CDC guidelines. If patients’ BMI is in group 4 or above, they are categorized as obese. Age refers to the age at which patients were diagnosed with COVID-19.

### Statistical analyses

Chi-squared analyses were used to check the association between each variable and COVID-19 death among cancer patients diagnosed with COVID-19. In addition, chi-squared analyses were used to compare the association between each variable and race. A log-rank test was performed to evaluate whether differences in COVID-19 survival exist between Non-Hispanic White and Non-Hispanic Black cancer patients.

Mediation analysis with Cox proportional hazard regression was conducted following the methods outlined in Yu et al.^43^. A variable is considered a mediator if it meets the criteria of being a risk factor for the outcome variable, associated with the predictor variable, and lies in the causal pathway between the predictor and outcome^43^. Confounders are variables that are not part of the causal pathway but may affect the relationship between predictor and outcome. The total effect is defined as the changing rate in the outcome variable when the predictor variable varies through all paths. The direct effect is the effect between the predictor and outcome variables after adjusting for all mediators. The indirect effect is the effect through an explanatory variable (mediator) between the predictor and response variables. Relative effects are reported as a percentage, indicating the proportion of the direct or indirect effect with respect to the total effect. It is calculated by dividing the direct or indirect effect by the total effect.

To evaluate the effect of comorbidities in explaining the relationship between race and COVID-19 mortality, two separate analyses were conducted. We first evaluated the primary effect of Charlson Comorbidity Index as a potential mediator between race and COVID-19 mortality, adjusting for sex, age, insurance, poverty, cancer type, SEER summary stage, obesity, age, and sex. The second analysis looked at each of the various diseases (e.g. MI, CHF, PVD, etc.) separately as mediators, again including previously mentioned demographic/clinical variables as covariates, to see how specific diseases contributed to explaining the observed racial differences in the COVID-19 death.

To evaluate which variables should be included as covariates, we first performed a screening by examining two associations. The first association looked at the significance of the relationship between race and each potential confounder using the Chi-squared test, given that all potential confounders were categorical variables. The second association used Type-III tests in the full Cox model to identify variables significantly contributing to the survival rate after adjusting for all potential variables. To prevent overlooking important variables during this screening process, we set the significance level at 0.1.

All analyses were done in R and the mediation analysis was performed using the mma package^43,44^. The mma package which was developed by Yu et al., uses both G-computation methods and nonlinear models to find conclusions about the relationship between predictor and outcome variables through various pathways^44,45^. This method allows for the simultaneous incorporation of multiple mediators or confounders, which results in more accurate estimations of mediation and confounding effects. Both linear and nonlinear predictive models were used in the mma package to help enhance traditional methods that consider linear associations only. To summarize, the mma package is a powerful tool for unveiling complex causal pathways in mediation analysis^46^.

## Results

In this study, our primary objective was to explore the effect of comorbidities in explaining the relationship between race and COVID-19 deaths amongst cancer patients. This study initially evaluated whether Charlson Comorbidity Index significantly mediated this relationship and then looked at individual diseases to analyze the specific effects of different diseases.

### Differences in survival

A significant difference in survival was seen between Non-Hispanic Black and Non-Hispanic White cancer patients, with Non-Hispanic Black patients having a worse survival rate than Non-Hispanic White patients (Log rank statistic = 21.9, *p* < 0.0001). The six-month survival rate of cancer patients were 0.940 and 0.916 for Non-Hispanic White and Non-Hispanic Black cancer patients respectively.

### Selection of covariates

Table 2 presents the summary statistics for each variable, including the mediators, and its association with race (P-value 1), along with the results of screenings for both analyses, with Charlson (P-value 2) and with all diseases included separately (P-value 3).

**Table 2:** Racial differences in socioeconomic factors and chronic diseases among cancer patients who tested positive for COVID-191 in 2020, Louisiana.

	Non-hispanic white patients with COVID-19 (*N* = 8826, %)	Non-hispanic black patients with COVID-19 (*N* = 3699, %)	*P*-value 1^6^	*P*-value 2^7^	*P*-value 3^8^
Sex					
Male	47. 64	43.85	0.0001	**< 0.0001**	**< 0.0001**
Female	52.36	56.15			
Age (year) ^2^					
20–49	11.78	13.81	< 0.0001	**< 0.0001**	**< 0.0001**
50–64	30.64	37.52			
	28.93	29.12			
75/+ 65–74	28.65	19.55			
Insurance ^3^					
Private	54.57	39.77	< 0.0001	**0.062**	**0.078**
Medicare/Other Public	30.95	28.74			
Medicaid	7.09	23.90			
No Insurance/Unknown	7.39	7.60			
Poverty^4^					
0%–< 10%	8.29	1.35	< 0.0001	**0.089**	**0.072**
10%–< 20%	21.38	7.03			
≥ 20%	43.83	25.91			
Unknown	26.49	65.71			
Cancer type					
Breast	20.75	25.82	< 0.0001	0.249	0.256
Prostate	15.52	20.82			
Colon and rectum	7.97	11.81			
Hematopoietic	4.15	4.79			
Lung and Bronchus	3.93	4.76			
Other	47.69	32.01			
SEER summary stage					
In situ	14.12	8.92	< 0.0001	**0.038**	**0.042**
Localized	55.80	55.99			
Regional	17.82	20.71			
Distant	9.70	12.01			
Unknown	2.57	2.37			
Obesity at cancer diagnosis					
No	53.21	43.00	< 0.0001	**0.053**	**0.032**
Yes	46.79	57.00			
Charlson Comorbidity Index					
0	80.51	69.21	< 0.0001	**< 0.0001**	N/A
1–2	17.35	25.52			
3/+	2.14	5.27			
Myocardial infarction					
No	99.41	98.70	< 0.0001	N/A	0.157
Yes	0.59	1.30			
Congestive heart failure (CHF)					
No	98.26	96.51	< 0.0001	N/A	0.166
Yes	1.74	3.49			
Peripheral vascular disease (PVD)					
No	98.54	98.16	0.142	N/A	**0.099**
Yes	1.46	1.84			
Chronic pulmonary disease (CPD)					
No	94.31	93.32	0.037	N/A	**0.054**
Yes	5.69	6.68			
Diabetes					
No	89.64	80.91	< 0.0001	N/A	0.601
Yes	10.36	19.09			
Renal disease					
No	97.95	95.08	< 0.0001	N/A	**0.008**
Yes	2.05	4.92			
Liver disease					
No	99.31	98.59	0.0002	N/A	**0.030**
Yes	0.69	1.41			
Other diseases					
No	97.78	95.97	< 0.0001	N/A	0.158
Yes	2.22	4.03			

Significant values are in [bold].

^1^Patients diagnosed with cancer between 2015 and 2019 and tested positive for COVID-19 in 2020.

^2^Age at the first positive COVID-19 test.

^3^Health insurance at cancer diagnosis and around the first course of treatment.

^4^Poverty at the residential census tract at the time of the first COVID-19 positive.

^5^Include dementia, paralysis, ulcers, rheum, AIDs, and cerebrovascular disease.

^6^P-Value 1: Chi-square test of the association between race and the row variable.

^7^P-Value 2: P-value of type III test for the row variable in the full Cox model including only Charlson Comorbidity Index.

^8^P-Value 3: P-value of type III test for the row variable in the full Cox model including each comorbidity.

In the first analysis, sex, age, poverty, obesity, insurance, and SEER summary stage were identified as covariates. Cancer type was shown to not be significantly associated with the risk of COVID-19 death adjusting for other covariates. However, based on our conceptual model, we forced in this variable as a potential mediator. In the second analysis, sex, age, poverty, summary stage, obesity, and insurance were identified as potential covariates. We again forced in all demographic/clinical variables in the conceptual model as potential mediators in the analysis. Note that cancer type is significantly related to the COVID-19 death in a univariate analysis (Table 3). The hazard ratio (HR) of 0.443 for Breast Cancer means that the hazard of death for patients with Breast Cancer is 55.71% lower compared to those with lung and bronchus cancer. Similarly, the hazard of death is 28.6%, 30.6% and 39.6% lower for prostate cancer, colon and rectum cancer and other cancer respectively compared to lung and bronchus cancer.

**Table 3:** Summary of hazard ratios and 95% confidence interval for cancer types.

Group	Hazard ratio	95% confidence interval	*P* Value
Breast cancer	0.443	(0.320, 0.613)	0.000
Prostate cancer	0.714	(0.520, 0.979)	0.037
Colon and rectum cancer	0.694	(0.489, 0.985)	0.041
Hematopoietic cancer	1.169	(0.808, 1.691)	0.407
Other cancer	0.604	(0.450, 0.811)	0.001

The univariate analysis showed that compared to lung and bronchus cancer, breast cancer, prostate cancer, colon and rectum cancer, and other cancers are significantly related to the hazard of dying from COVID-19. We further performed mediation analyses stratified by each cancer type to identify whether the Charleston Comorbidity Index (CCI) can partially explain the relationship observed between race and COVID-19-related death, adjusting for all the covariates. Table 4 showed that there were significant racial differences in the COVID-19 deaths for prostate cancer and other cancers separately. CCI can partially explain the racial differences in death rates for other cancer type.

**Table 4:** Summary of mediation effect estimations in hazard ratios stratified by cancer type.

Models by cancer type	Lung cancer	Breast cancer	Prostate cancer	Colon and rectal cancer	Other cancer
Mediator: CCI					
Indirect effect (95% CI)	0.044 (− 0.220, 0.535)	0.173 (− 0.003, 0.521)	0.093 (− 0.009, 0.353)	0.018 (− 0.113, 0.220)	0.280 (0.088, 0.575)
Direct effect	0.492 (− 0.754, 3.454)	0.162 (− 0.758, 1.591)	1.241 (0.285, 2.889)	− 0.007 (− 0.791, 1.073)	1.828 (0.696, 3.268)
Total effect	0.528 (− 0.688, 3.635)	0.318 (− 0.607, 1.91)	1.327 (0.380, 3.073)	0.001 (− 0.799, 1.115)	2.109 (0.902, 3.702)

### Charlson comorbidity index in explaining the differences in COVID-19 deaths

For the first analysis, including only the Charlson Comorbidity Index, Table 5 shows the estimated direct, indirect, and relative effects explaining the observed difference in COVID-19-related death among cancer patients using the linear (Cox proportional hazard model) model. A relative effect is defined as the corresponding direct or indirect effect divided by the total effect.

**Table 5:** Summary of mediation/confounding effect estimations and hazard ratios for analysis 1.

Mediator/confounder	Indirect effect (95% CI)	Relative effect (%) (95% CI)	HR^1^ (95% CI)	*P*-value
Charlson Comorbidity Index	0.14 (0.07, 0.24)	12.72 (6.32, 21.37)	1.15 (1.07, 1.27)	< 0.0001
Direct effect	1.01 (0.52, 1.58)	87.28 (78.33, 93.82)	2.75 (1.68, 4.85)	< 0.0001
Total effect	1.15 (0.63, 1.76)		3.16 (1.88, 5.81)	< 0.0001

^1^HR: hazard ratio.

Compared with Non-Hispanic White patients, Non-Hispanic Black cancer patients have an average higher hazard rate (Total Effect (TE) = 1.15, CI = 0.63–1.76, see Table 5). The COVID-19 death hazard rate amongst Non-Hispanic Black cancer patients is 3.16 times, or 216% higher than, that for Non-Hispanic White cancer patients (e^1.15^ = 3.16). Direct effect (DE) is the observed difference that cannot be explained by the Charlson comorbidity index, which is estimated as 1.01 (CI = 0.52–1.58). Because the direct effect was significantly different from 0, the racial differences in the risk of COVID-19 death amongst cancer patients cannot be completely explained by the index. Specifically, 87.28% (1.01/1.15) of the racial differences in the risk of COVID-19 death cannot be explained by the index after adjusting for the confounders/covariates. Charlson Comorbidity Index explained 12.72% of the difference in the risk of COVID-19 death. The effect is significant (p-value < 0.0001).

**Fig. 3:**
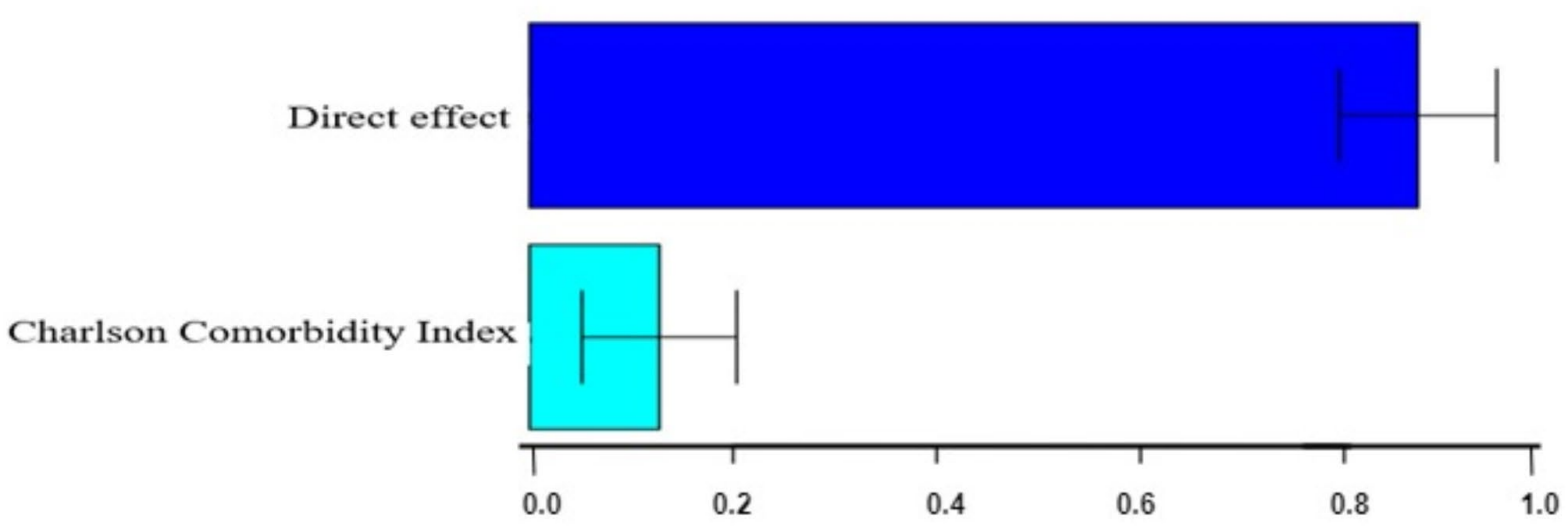
Relative effects from Analysis 1. It shows the estimated relative effects and confidence intervals based on the mediation analysis using the Cox proportional hazard model.

**Fig. 4:**
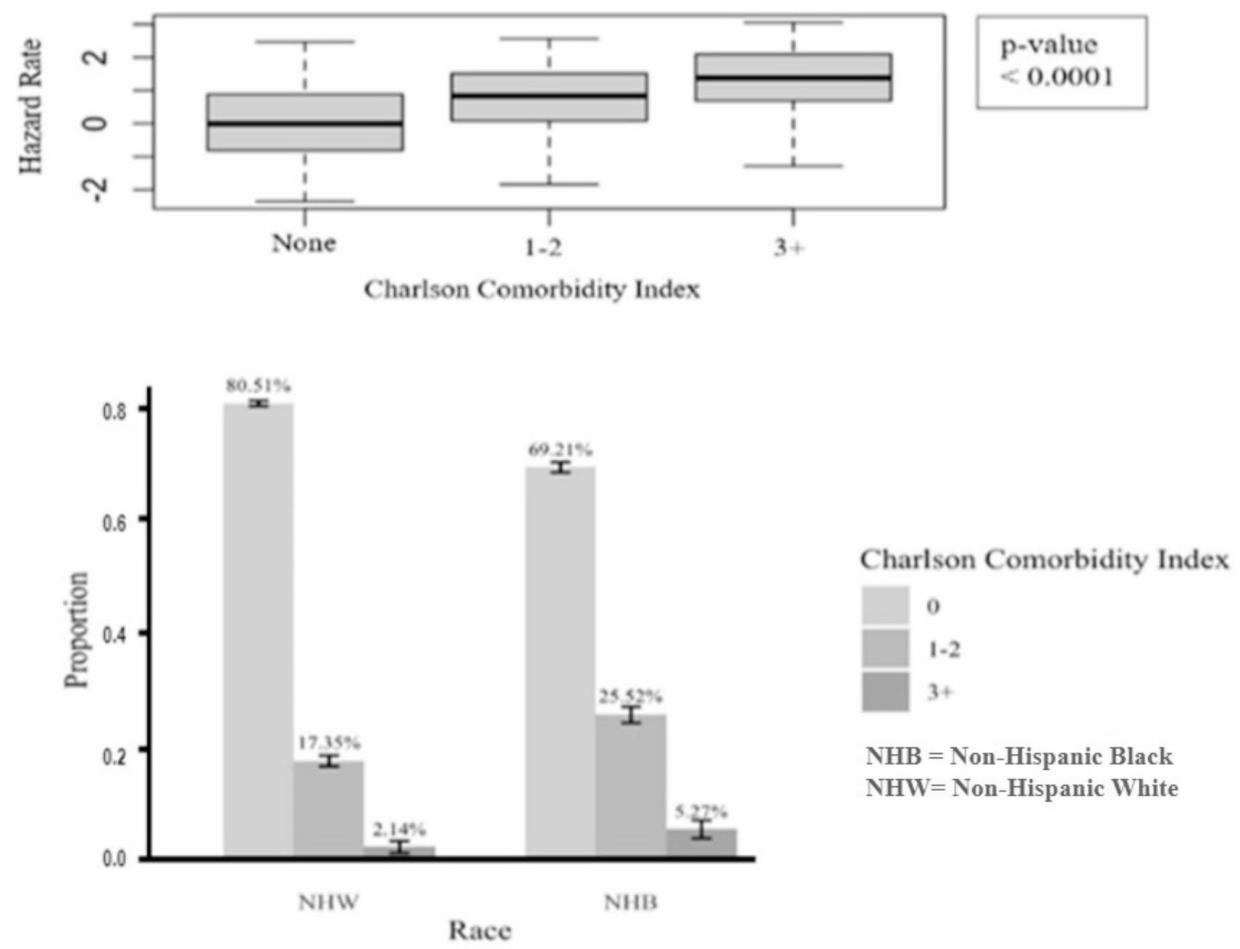
Indirect effect of Charlson Comorbidity Index on COVID-19 related death and Charlson group proportions in Analysis 1.

Figure 3 shows the estimated relative effects and confidence intervals based on the mediation analysis using the Cox proportional hazard model. Figure 4 shows that individuals with a Charlson score of 3 or higher have a higher risk of COVID-19 death, while there is a higher proportion of Non-Hispanic Black patients with a score of 3+.

### Individual comorbidity in explaining variations in health outcomes

In the second analysis, each disease is included in the mediation analysis to assess their individual contribution to the relationship between race and COVID-19-related death amongst cancer patients. Table 6 shows the estimated direct and indirect effects, as well as the relative effect, using the linear (Cox proportional hazard model) model.

**Table 6:** Summary of mediation/confounding effect estimations for analysis 2.

Mediator/confounder^1^	Indirect effect (95% CI)	Relative effect (%)	*P*-value
MI	− 0.01 (− 0.03, 0.01)	− 0.58 (− 2.64, 0.58)	0.404
CHF	0.02 (− 0.01, 0.06)	1.86 (− 1.13, 5.73)	0.209
PVD	0.02 (− 0.01, 0.04)	0.87 (− 0.66, 3.29)	0.307
CPD	0.00 (− 0.02, 0.02)	0.19 (− 1.77, 2.15)	0.776
Diabetes	0.02 (− 0.05, 0.09)	1.50 (− 4.65, 7.78)	0.615
**RD**	**0.05 (0.01**,** 0.11)**	**4.49 (0.82**,** 10.24)**	**0.015**
LD	0.02 (0.00, 0.06)	1.81 (− 0.16, 5.53)	0.082
Other	0.02 (− 0.01, 0.06)	1.74 (− 0.89, 5.41)	0.187
Direct effect	**1.00 (0.53, 1.57)**	**89.50 (80.12, 97.23)**	**< 0.0001**
Total effect	**1.12 (0.57, 1.71)**		**< 0.0001**

Significant values are in [bold].

^1^MI = myocardial infarction, CHF = chronic heart failure, PVD = peripheral vascular disease, CPD = chronic pulmonary disease, RD = renal disease, LD = liver disease, Other = other diseases.

**Fig. 5:**
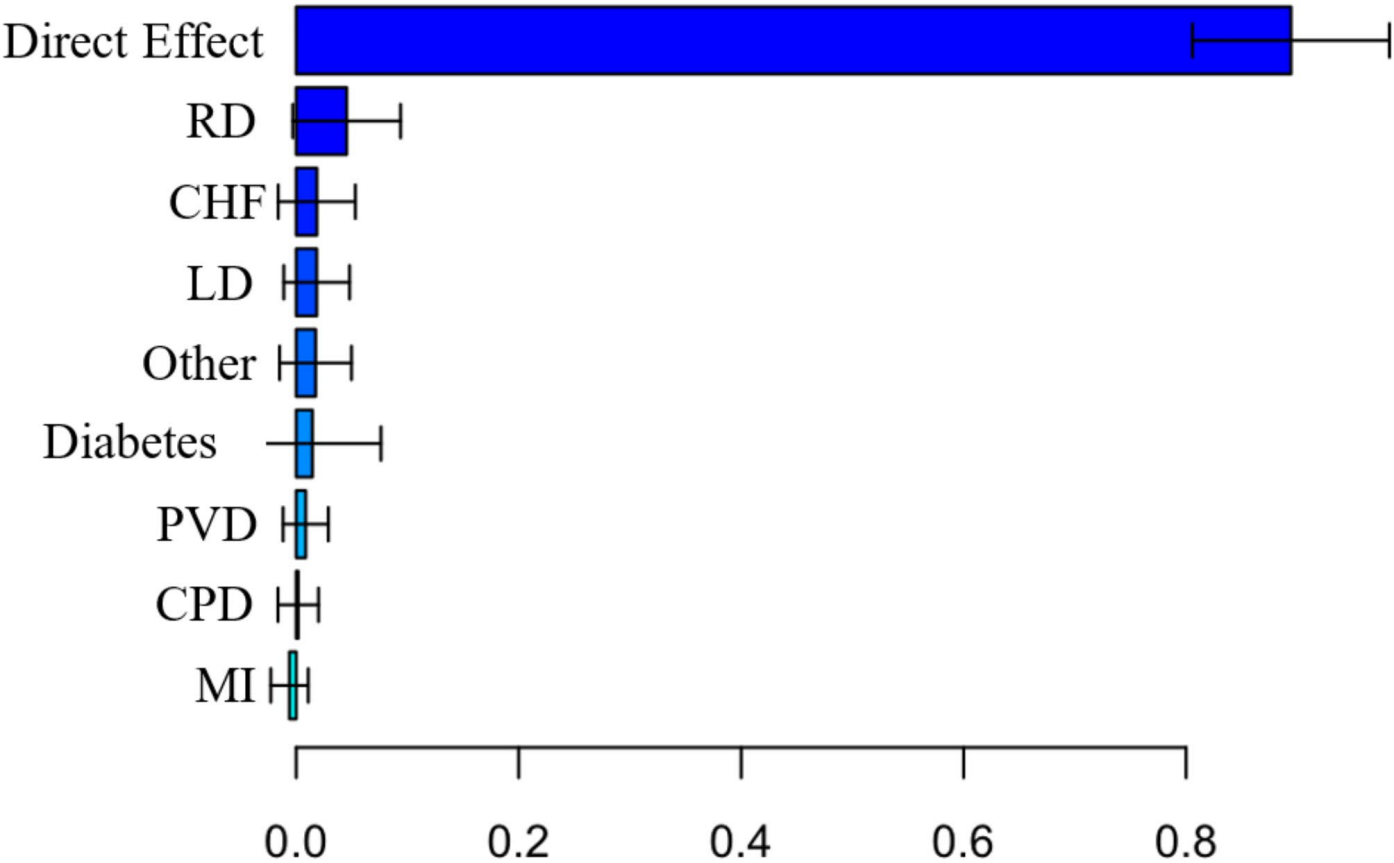
Relative effects from Analysis 2. It shows the estimated relative effects and their confidence intervals for individual diseases. RD = Renal Disease, CHF = Chronic Heart Failure, LD = Liver Disease, Other = Other Diseases, PVD = Peripheral Vascular Disease, CPD = Chronic Pulmonary Disease, MI = Myocardial Infarction.

**Fig. 6:**
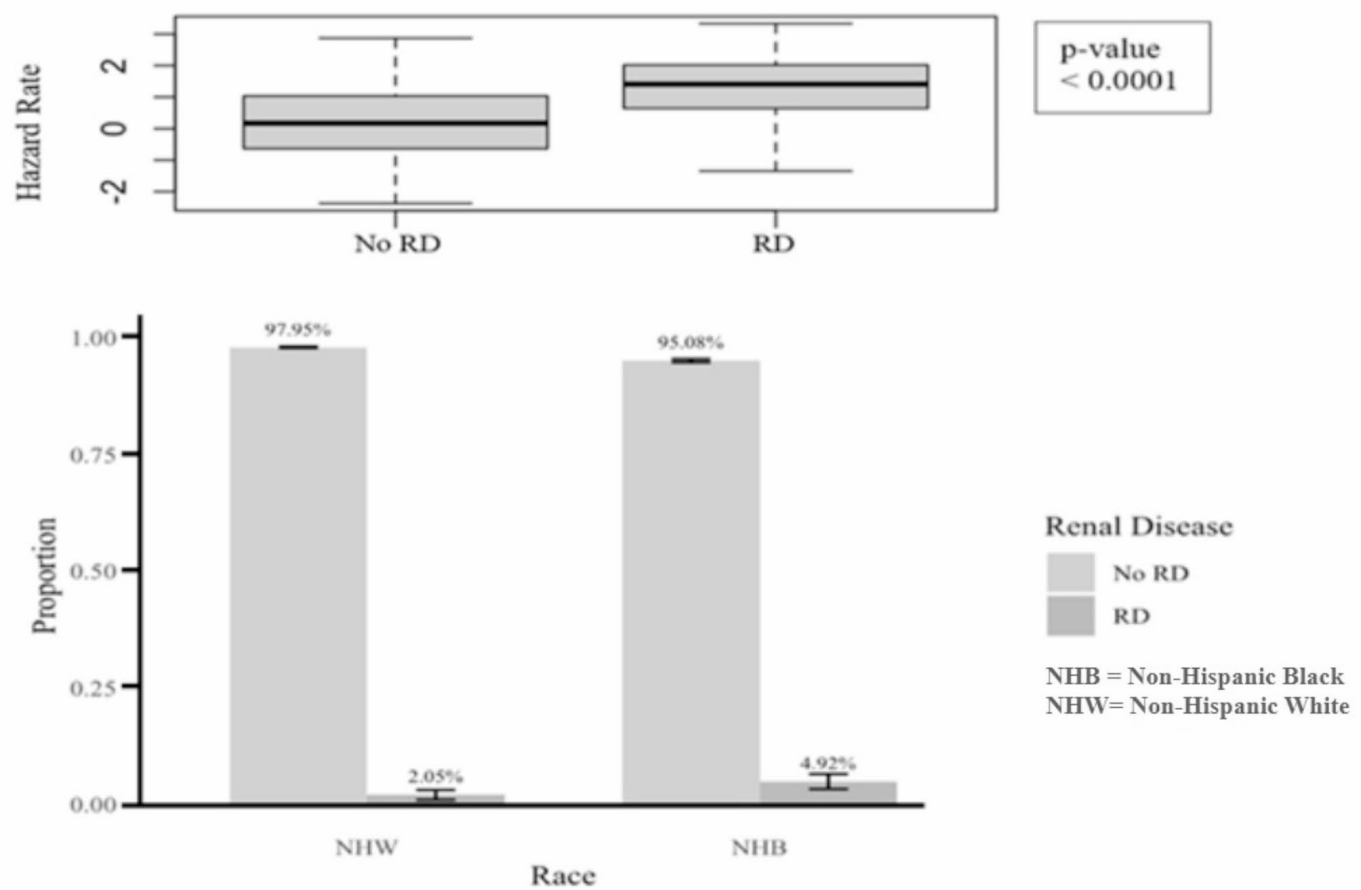
Indirect effect of renal disease on COVID-19 related death and renal disease proportions in Analysis 2.

Similar to the analysis with Charlson index, Non-Hispanic Black cancer patients have an average higher risk of COVID-19 death than Non-Hispanic White cancer patients (Total Effect (TE) = 1.12, CI = 0.61–1.71, see Table 5). The direct effect (DE) was once again significantly different from 0, indicating that the racial difference in the risk of COVID-19 death cannot be completely explained by all included mediators (DE = 1.00, CI = 0.53–1.57). Renal disease significantly contributed to the observed difference, explaining 4.49% of the difference in COVID-19 related death (*p* < 0.0001). Figure 5 shows the estimated relative effects and their confidence intervals. Figure 6 shows that cancer patients with renal disease have a higher risk of COVID-19 related death, and there is a larger proportion of Non-Hispanic Black patients with renal disease.

## Discussion

Mediation modeling serves as a valuable tool in the field of public health aiding in the comprehensive explanation of established relationships between predictors and outcomes. By incorporating various variables that could potentially mediate or elucidate the established relationship, mediation analysis offers deeper insights into the underlying mechanisms at play. In this paper, mediation analysis methods were used to explore the relationship between race and COVID-19 related death amongst cancer patients. Two separate analyses were performed—one including Charlson Comorbidity Index as the primary mediator and the other including a number of individual diseases. In both analyses conducted, Non-Hispanic Black cancer patients exhibited a consistently higher average hazard rate compared to Non-Hispanic White cancer patients. The first analysis revealed that the Charlson Comorbidity Index was a significant mediator in explaining the association. In addition, individuals with a Charlson score of 3 or higher have a higher risk of COVID-19 death consistent with previous research^21–23^.

The second analysis showed that specifically, renal disease emerged as the primary contributor to the racial difference. COVID-19 related death amongst renal disease patients may be high due to death rate associated with pulmonary infection in renal disease patients^47^. Renal disease, also known as kidney disease, indicates sudden loss or damage to kidney functions. Renal disease patients have increased inflammatory immune response, making them more susceptible to severe infections. Recent studies have shown that COVID-19 patients experience a more rapid decline in kidney function compared to others who had pneumonia from other causes^52,53^. Mostly due to the high expression of angiotensin-converting enzyme 2 (ACE2) receptor in the kidney through which the virus enters the cells^53^. African Americans are 3–4 times more like to have kidney failure compared to Caucasian Americans due to various social, economic and environmental factors^54,55^. This study emphasizes the need for healthcare providers to prioritize early detection and effective management of chronic diseases, especially renal disease, in Black communities that are at a higher risk of severe COVID-19 outcomes^32,33^.

While the current research serves as a valuable initial exploration into the mediation of the relationship between race and COVID-19-related death among cancer patients, it is important to acknowledge that the current study does not provide a comprehensive explanation for the observed difference in the risk of COVID-19 death among cancer patients. Moreover, the study focused on overall COVID-19 survival among cancer patients to provide a comprehensive understanding of how cancer as a general condition impacts COVID-19 outcomes. While survivorship outcomes vary significantly across cancer types, the overarching goal of this study was to explore the shared vulnerabilities faced by cancer patients which are likely to influence COVID-19 survival regardless of the specific type of cancer.

There are a few limitations in this study. One in particular concern the availability of the poverty variable. For this study, we used poverty at the time of patients’ COVID-19 diagnosis. While our study lacked information on socioeconomic variables post-diagnosis, previous studies suggest that they are relatively stable indicators for most individuals^50,51^. However, it may be more beneficial to use poverty recorded during patients’ cancer diagnosis. This may prevent the excess of missing data that was seen for this variable. In addition, the insurance variable was recorded at the time of cancer diagnosis, which has the potential to change by the time a patient is diagnosed with COVID. Overall, the use of the most updated and accurate demographic variables would help to improve the analysis.

A second limitation is that this study did not extensively explore the progression of patients’ cancer. How long a patient has had cancer can impact on the severity of the cancer, a factor that was not explored in these analyses. Future analyses should include how long a patient has had cancer as a potential covariate. In addition, the complexity of the COVID-19 pandemic was simplified in this study. However, it is important to explore the different variants of COVID-19 (alpha, beta, delta, and omicron), as well as the timing of the peaks and the introduction of vaccines.

Additionally, the cause of death data extends only until December 2021 for patients who tested positive for COVID-19 from the beginning of the pandemic in 2020 through June 30, 2021. Because of the limited follow-up time, the impact of COVID-19 infection on long-term COVID-19 death is unknown.

Lastly, significant changes might have occurred in COVID-19 variants and vaccination rates during the one-year study period. Our dataset did not include information on these factors, which could have influenced mortality outcomes. In future research, we plan to use the time period as a proxy variable to better capture the effects of different COVID-19 variants and vaccination rates. Additionally, we aim to expand the scope of the study to include a nationwide cancer population to increase the sample size and improve the generalizability of our findings.

Overall, this study is important in establishing the relationship between race and COVID-19-related deaths. Comorbidities, particularly renal disease, were shown to partially explain this observed difference. The results of this study have the potential to aid in the development of interventions that help to reduce the differences in the risk of COVID-19 death. By focusing on treating and managing chronic diseases, particularly renal disease, medical practitioners may be able to narrow the gap in COVID-19-related mortality among affected populations.

## Electronic supplementary material

Below is the link to the electronic supplementary material.

Supplementary Material 1

## Data Availability

The data that support the findings of this study is restrictedly accessible by requesting from the Louisiana Tumor Registry and will not be released without IRB approval. For inquiries regarding the data, researchers are encouraged to reach out to Lauren Maniscalco at lspiza@lsuhsc.edu for further details on the LTR’s data release policies.
