# Supplementary material for: Differences in Covid-19 deaths amongst cancer patients and possible mediators for this relationship
Source: Sci Rep. 2025 Mar 26;15:10407. doi: 10.1038/s41598-025-95037-3 (PMC11947243; doi:10.1038/s41598-025-95037-3)
Supplement: Supplementary file 1 — Supplementary Material 1 [file 41598_2025_95037_MOESM1_ESM.pdf]

TITLE: Racial Disparities in Covid-19 Deaths Amongst Cancer Patients and Possible Mediators for this Relationship

Authors: Leah Vaidya, MS[1]; Nubaira Rizvi, MS[1]; Xiao-Cheng Wu, MD, MPH[2]; Lauren S. Maniscalco, MPH[2]; Yong Yi, MS, PhD[2]; Augusto Ochoa, MD[3]; Qingzhao Yu, PhD[1]*

[1] Biostatistics and Data Science, School of Public Health, LSU Health Sciences Center, USA
[2] Louisiana Tumor Registry, School of Public Health, LSU Health Sciences Center, USA
[3] Stanley S. Scott Cancer Center, School of Medicine, LSU Health Sciences Center, USA

Leah Vaidya, lvaidy@lsuhsc.edu, School of Public Health, Louisiana State University Health Sciences Center New Orleans, LA, USA

Nubaira Rizvi, nrizvi@lsuhsc.edu, School of Public Health, Louisiana State University Health Sciences Center New Orleans, LA, USA

Xiao-Cheng Wu, xwu@lsuhsc.edu, School of Public Health, Louisiana State University Health Sciences Center New Orleans, LA, USA

Lauren S. Maniscalco, lspiza@lsuhsc.edu; Louisiana Tumor Registry, School of Public Health, Louisiana State University Health Sciences Center New Orleans, LA USA

Yong Yi, yyi@lsuhsc.edu; Louisiana Tumor Registry, School of Public Health, Louisiana State University Health Sciences Center New Orleans, LA, USA

Augusto Ochoa: AOchoa@lsuhsc.edu; Stanley S. Scott Cancer Center, School of Medicine, Louisiana State University Health Sciences Center, USA

Corresponding author: Qingzhao Yu, Qyu@lsuhsc.edu, School of Public Health, Louisiana State University Health Sciences Center New Orleans, LA, USA

R codes for the main models:

```
library(mma)

# Model 1 with just Charlson as mediator, everything else is a covariate
y=Surv(data$TimeDiff, data$CovDeath)  # outcome: survival time and censor variable
x= data[,c(3:10)]    #x is all potential confounders and mediators
pred=data[,1]    # race

mma.b.b.glm<-mma(x,y,pred=pred,catmed=c(8), catref=c( 0), predref=1, alpha=1,
alpha2=1, n=2, n2=5000)

summary(mma.b.b.glm, quant=TRUE)


# Model 2 with each disease as mediator, everything else is a covariate
y=Surv(data2$TimeDiff_final,data2$CovDeath) )  # survival outcome
 x=data2[,c(3:17)]   #x is all potential confounders and mediators
 pred=data2[,1]   #race

 mma.b.b.glm1<-mma(x,y,pred=pred, binmed =c(6:13), binref=c(0, 0, 0, 0, 0, 0, 0, 0),
predref=1, alpha=1, alpha2=1, n=2, n2=5000)

summary(mma.b.b.glm1, quant=TRUE)

plot(mma.b.b.glm1,vari="renaldisease")
```
